# A population of dermal Langerin^+^ dendritic cells promote the inflammation in mouse model of atopic dermatitis

**DOI:** 10.3389/fimmu.2022.981819

**Published:** 2022-10-03

**Authors:** Chunying Xiao, Zhenlai Zhu, Chen Zhang, Jixin Gao, Yixin Luo, Hui Fang, Hongjiang Qiao, Wei Li, Gang Wang, Meng Fu

**Affiliations:** ^1^ Department of Dermatology, Xijing Hospital, Fourth Military Medical University, Xi’an, China; ^2^ Department of Dermatology, Huashan Hospital, Fudan University, Shanghai, China

**Keywords:** atopic dermatitis, dendritic cell, dermal Langerin^+^ dendritic cells, Langerhans cells, thymic stromal lymphopoietin

## Abstract

Cutaneous dendritic cells (DCs) have been implicated in the pathogenesis of atopic dermatitis (AD). However, the specific role of different subsets of DCs has not been well defined. This study aimed to investigate the contributions of Langerhans cells (LCs), resident dermal Langerin^+^ DCs (r-Langerin^+^ dDCs), and newly infiltrated inflammatory dermal Langerin^+^ DCs (i-Langerin^+^ dDCs) in an AD mouse model induced by the topical application of MC903. The result showed that depletion of i-Langerin^+^ dDCs in DTR mice after multiple diphtheria toxin (DT) injection significantly reduced thymic stromal lymphopoietin (TSLP) production in lesions and skin inflammation alleviation. However, depletion of LCs or r-Langerin^+^ dDCs didn’t resulted in significant changes in skin inflammation of DTA or single DT injection-treated DTR mice compared with the wild-type (WT) mice. DT-treated DTR-WT chimeric mice with the depletion of bone marrow (BM)-derived i-Langerin^+^ dDCs resulted in markedly decreased skin inflammation than controls, while PBS-treated chimeric mice (DTR-WT) with only the depletion of r-Langerin^+^ dDCs showed inflammation comparable to that in WT mice. Furthermore, TSLP contributed to the upregulation of Langerin expression in BM-derived DCs and promoted the maturation of Langerin^+^ DCs. In summary, the present study demonstrated that the newly infiltrated inflammatory dermal Langerin^+^ DCs were essential for AD development and local TSLP production, and TSLP further promoted the production of BM-derived i-Langerin^+^ dDCs, which might maintain AD inflammation.

## Introduction

Atopic dermatitis (AD) is a pruritic, chronic, relapsing inflammatory skin disorder with an increasing prevalence in developing countries. It is characterized by intense scratching, increased serum IgE levels, and a helper T (Th) 2-dominated systemic and local immune response to cutaneous introduced antigens. A combination of genetic factors, environmental triggers, and skin barrier dysfunction contributes to the initiation of AD; both innate and adaptive immune systems are involved in the dysregulated immune responses (1, 2).

Dendritic cells (DCs) are antigen-presenting cells with an important role in the innate and adaptive immune systems. In the steady state, three major cutaneous DC populations are present according to the Langerin expression and their location: Langerhans cells (LCs) specifically expressing Langerin in the epidermis, dermal Langerin^+^ DCs, and dermal Langerin^-^ DCs (3). During inflammation, an additional population of myeloid DCs “inflammatory DCs” infiltrate into the dermal compartment of skin lesions (4). In human atopic lesions, an overall increase in the number of DCs has been found in the epidermis and dermis. However, little is still known about the roles of the different DC subsets in the pathogenesis of AD. Accumulating studies have indicated that DCs are involved in the induction of Th2 response and implicated in Th2-mediated diseases, such as AD, helminth infections, and allergic airway inflammation (5–8). Current evidence indicates that the immune responses initiated by DC populations are complex and dependent on the local microenvironment. TSLP is highly expressed in the epidermis of atopic skin and has been recognized as a key epithelium-derived cytokine participating in the initiation of AD (9, 10). Recent findings show that DC subsets can respond to TSLP, participate in different T cell-mediated immune responses, and are involved in the pathogenesis of the disease (11). An *in-vitro* study showed that blood CD11c^+^ immature DCs in humans could be activated by TSLP and prime Th cells to become Th2 cytokine−producing cells. Several studies found that epidemic LCs responded to TSLP and induced Th2 polarization *in vitro* and in mice. In addition, populations of EpCAM^hi^ DCs, including LCs acted upon by TSLP, could migrate to skin-dLNs to elicit type 1/type 17 and type 2/type 9 cytokine production (12). Shino et al. reported that plasmacytoid DCs, mostly Langerin^-^ DCs activated by TSLP, could induce the generation of Foxp3^+^ regulatory T cells, but did not induce a Th1/Th2 response. The latest study also confirmed that plasmacytoid DCs suppressed Th2 responses induced by epicutaneous sensitization, which was the most important sensitization route for AD (13). Dermal Langerin^+^ DCs, a minor population of skin DCs, also expressed Langerin but were totally distinguished from epidemic Langerin^+^ LCs. Studies showed that dermal Langerin^+^ DCs played important roles in various immune responses. However, less attention was paid to the role of dermal Langerin^+^ DCs, including resident dermal Langerin^+^ DCs (r-Langerin^+^ dDCs) and new infiltrated inflammatory dermal Langerin^+^ DCs (i-Langerin^+^ dDCs) in the pathogenesis of AD, as well as the effect of TSLP on the populations of DCs.

In the present study, we characterized the roles of LCs, r-Langerin^+^ dDCs, and i-Langerin^+^ dDCs in MC903-induced AD-like dermatitis using hLangerin-DTA and mLangerin-DTR mice. We found that i-Langerin^+^ dDCs derived from the bone marrow (BM) are critical for the pathogenesis of AD, while LCs and r-Langerin^+^ dDCs are dispensable for the disease. A high level of TSLP in local atopic lesions can promote the formation of BM-derived i-Langerin^+^ dDCs by upregulating the expression of Langerin. Our study was novel in distinguishing the three Langerin^+^ DC populations and determining their specific role in AD, which further enriched the knowledge on the roles of DC subsets in the immunopathogenesis of the disease.

## Materials and methods

### Mice and treatment

hLangerin-DTA (DTA), mLangerin-DTR (DTR) were purchased from the Jackson Laboratory. All mice were 8 to 12 weeks old and maintained on a 12-hour light/dark cycle under specific pathogen-free conditions. All animal experiments were performed in compliance with the guidelines outlined in the Guide for the Care and Use of Laboratory Animals of the University. For production of AD model in mice, 2 nmol MC903 (calcipotriol; Leo Pharma, Ballerup, Denmark) was topically painted on mouse ears for 14 consecutive days. For ovalbumin (OVA)-specific antibodies in percutaneous sensitization assay, 50 ug OVA was applied locally on both ears every the other day with concomitant MC903 treatment.

At the indicated time points, ear thickness was measured with a microcalliper (Type 0-12.7, YUEQING JINGCHENG, Shanghai, P. R. China). Scratching was monitored by counting the number of scratches for a period of 30 minutes. Trans-epidermal water loss (TEWL) was measured on the ear using Tewameter TM300 probes in the Courage & Khazaka MPA10 system and was recorded. Skin tissue was either snap-frozen for RNA, fixed in formalin for histopathologic analysis, or digested to achieve single-cell suspensions for further fluorescence-activated cell sorting (FACS) analysis. Blood samples were collected at indicated time and serum total IgE, OVA-specific IgG1, IgG2a and TSLP were analyzed by ELISA according to the manufacturer’s protocol.

### Cell isolation and flow cytometry

Ear tissues were minced with scissors and digested with 2 mg/ml collagenase type 11, 500 µg/ml hyaluronidase and 100 µg/ml DNase (all from Sigma-Aldrich) at 37°C for 40 minutes. Cell suspensions were then filtered through a 200-mesh strainer and harvested for antibody staining. The single-cell suspension was firstly incubated with anti-CD16/32 antibody (clone 93, Biolegend) to block Fc-receptors and then stained with fluorescent antibodies for 30 min. The antibodies used were all purchased from Biolegend (San Diego, CA, USA) unless indicated: CD4 (clone GK1.5, Biolegend), Gr-1 (clone RB6-8C5), CD8 (clone 53-6.7), CD11b (clone M1/70) and TSLPR (clone 22H9). Dead cells were excluded by staining with 7-aminoactinomycin Viability Staining Solution (7AAD, Biolegend). The recovered number in one ear was calculated using Count Bright™ Absolute Counting Beads (Invitrogen, USA). Flow cytometry was performed on a BD FACS Calibur instrument (BD Biosciences, USA), and data was analyzed using FlowJo 7.6.4 software (Treestar).

### Bone marrow transplantation

Single-cell suspensions of BM from the femurs were prepared from DTR or WT mice, and 5×10^6^ BM cells were injected into the tail vein of each lethally irradiated (9.0 gray) recipient mice of WT mice or DTR mice. And DTR-WT and WT-DTR chimeric mice were established, respectively. After 6 weeks, the chimeric mice were applied to produce MC903-induced dermatitis as we described above.

### Real-time quantitative reverse transcription polymerase chain reaction

Total RNA from ear skin tissues was extracted using TRIzol reagent (Invitrogen, Life Technologies, Carlsbad, CA, USA). Quantitative real-time polymerase chain reaction analysis (qRT-PCR) was conducted using SYBR Premix Ex Taq™ II (TaKaRa, Dalian, China) on a CFX384 Real-time PCR detection system (Bio-Rad, Hercules, CA, USA). Fold changes were normalized to those of HPRT (*hypoxanthine-guanine phosphoribosyltransferase*). All primers listed as follows (**Table 1**), were synthesized by Sangon Biotech (Shanghai, China).

**Table 1:** The sequences of primers.

IL-1β	Forward	5’-GCCACCTTTTGACAGTGATG-3’
	Reverse	5’-AAGGTCCACGGGAAAGACAC-3’
IL-4	Forward	5’-AGAAGGGACGCCATGCAC-3’
	Reverse	5’-GCATCGAAAAGCCCGAAAGA-3’
IL-6	Forward Reverse	5’-TGCAAGAGACTTCCATCCAGT-3’ 5’-CTGCAAGTGCATCATCGTTGT-3’
IL-10	Forward Reverse	5’-AGCCGGGAAGACAATAACTG-3’ 5’-CATTTCCGATAAGGCTTGG-3’
IL-13	Forward	5’-AGGAGCTGAGCAACATCAC-3’
	Reverse	5’-GGGTCCTGTAGATGGCATTG-3’
IFN-γ	Forward Reverse	5’-ACTGGCAAAAGGATGGTGAC-3’ 5’-ACCTGTGGGTTGTTGACCTC-3’
TSLP	Forward Reverse	5’-AGGGGCTAAGTTCGAGCAAA-3’ 5’-CGTCATTTCTCTCAGTTTCAGGG-3’
CXCL2	Forward Reverse	5’-CAACCACCAGGCTACAGGG-3’ 5’-GTTAGCCTTGCCTTTGTTCAGT-3’
TNF-α	Forward	5’-TAGCCCACGTCGTAGCAAAC-3’
	Reverse	5’-TAGCAAATCGGCTGACGGTG-3’
Langerin	Forward Reverse	5’- ATTCTGGAGATGGTGGCT -3’ 5’- TCCCTGCTTTGGTAAGTC -3’
HPRT	Forward Reverse	5’- AGTCCCAGCGTCGTGATTAG -3’ 5’- TTTCCAAATCCTCGGCATAATGA -3’

### BM-derived DCs (BMDCs) culture and treatment

BM cells were prepared from the femurs then cultured for 8 days in complete RPMI-1640 medium supplemented with 10% FCS, 225 U/ml GM-CSF (PeproTech, Rocky hill, New Jersey, USA), 2 ng/ml IL-4 (PeproTech), and/or 50 ng/mL TSLP (R&D Systems), and/or 10 ng/mL TGF-β (PeproTech). At day 9, cells were respectively lysed in TRIzol Reagent and RIPA, and subjected for qRT-PCR and Western Blot.

### Statistical analysis

All the data in our study were obtained from at least three independent experiments. Statistical analyses of the data were performed using Student’s un-paired two-tailed t test or two-way ANOVA analysis built into GraphPad Prism (GraphPad Software 8.0; San Diego, USA). All the data are presented as the mean ± standard error of the mean. *P* values < 0.05 were considered statistically significant.

## Results

### Inflammatory dermal Langerin^+^ DCs, but not resident dermal Langerin^+^ DCs or LCs, are necessary for MC903-induced AD-like dermatitis

**Figure 1:**
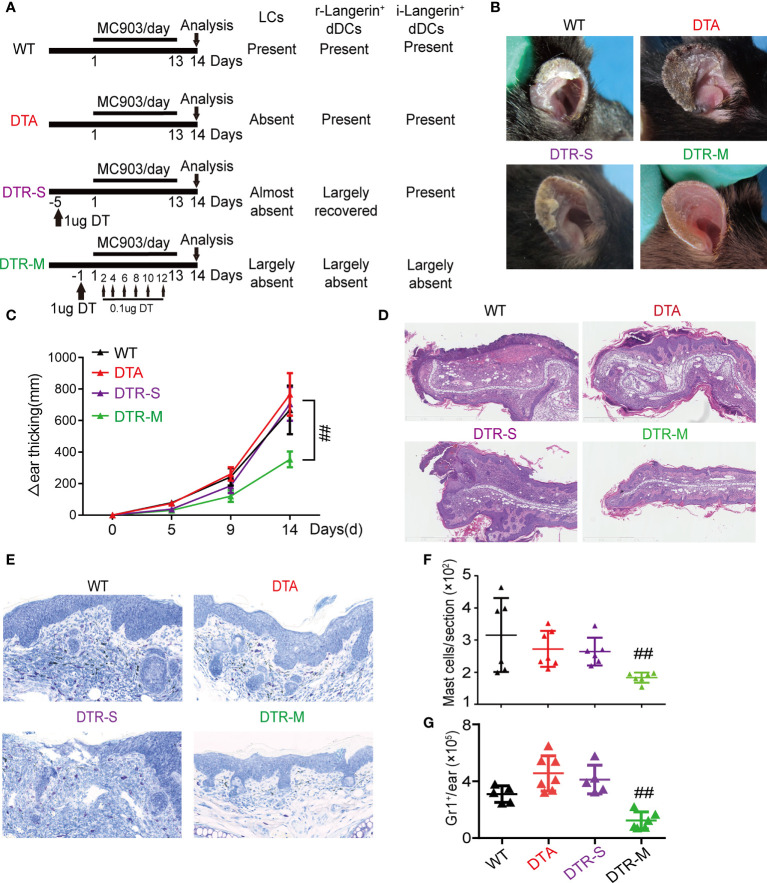
Inflammatory dermal Langerin^+^ DCs, instead of resident dermal Langerin^+^ DCs or LCs promote MC903-induced AD-like dermatitis. **(A)** Wild type (WT) mice, hLangerin-DTA (DTA) mice with lack of LCs, mLangerin-DTR (DTR) mice with DT injection for one time (DTR-S) and multiple times (DTR-M) were painted with MC903 on the ear for consecutive 14 days. **(B)** Gross appearance of MC903-treated ear at day 14. **(C)** Thickness increase of MC903-treated ear in each group. **(D)** Representative H&E-staining sections of lesion at day 14. Scale bar=1mm. **(E)** Representative toluidine blue revealing mast cell infiltration. Scale bar = 300 μm. **(F)** The absolute number of mast cells per section. **(G)** The absolute numbers of Gr1^+^ cells pre ear. One out of at least three representative experiments is depicted (mean ± SEM of n ≥ 3 mice per group). ^##^ *P* < 0.05 vs. WT, DTA and DTR-S groups. Langerhans cells, LCs; r-Langerin^+^ dDCs, resident dermal Langerin^+^ DCs; i-Langerin^+^ dDCs, inflammatory dermal Langerin^+^ DCs.

We used two transgenic mice, DTA and DTR mice, to determine the specific roles of LCs and dermal Langerin^+^ DCs. We termed resident dermal Langerin^+^ DCs as r-Langerin^+^ dDCs and new infiltrated dermal Langerin^+^ DCs as i-Langerin^+^ dDCs in the following text. LCs were constitutively absent in DTA mice due to the limited Langerin expression in epidermal LCs, while r-Langerin^+^ dDCs were unaffected (14). In DTR mice, both LCs and r-Langerin^+^ dDCs were inducible by DT injection. Prior studies demonstrated that the two Langerin^+^ DC subsets were reconstituted with different kinetics, and dermal Langerin^+^ dDCs recovered more rapidly. Different strategies of DT injection were used in DTR mice depending on the relapse gap. In DTR-S mice, after single DT injection 5 days prior to MC903 application, i-Langerin^+^ DCs were largely recovered to replace r-Langerin^+^ dDCs, while LCs were still ablated. In the DTR-M group (DTR mice with DT injection every other day multiple times), both LCs and r-/i-Langerin^+^ dDCs were ablated throughout the MC903 treatment. The protocol of the MC903-induced AD-like dermatitis mouse model is depicted in **Figure 1A**. The phenotype showed that DTR-M mice lacking both LCs and r-/i-Langerin^+^ dDCs had the lowest degree of inflammation than the other three groups, evidenced by the fewest scales, crusts, and minimum ear thickness increment. DTA mice and DTR-S mice, both lacking LCs, had inflammation similar to that in WT mice, with no significant difference in scales and thickness. Notably, no significant inflammation was observed in DTA and DTR-S mice, despite a discrepancy of r-Langerin^+^ dDCs (**Figures 1B, C**). Ear skin biopsies were taken on day 15, and hematoxylin and eosin staining demonstrated significant decreased epidermal thickness and less inflammatory cell infiltration in the ears of DTR-M mice, with no significant difference among DTA mice, DTR-S mice, and WT mice (**Figure 1D**). Mast cells and Gr1^+^ neutrophils, which are the characteristic inflammatory cells infiltrated in the atopic lesions, were detected by toluidine blue staining in skin sections and Gr1^+^ staining following flow cytometry analysis. The results showed that the numbers of mast cells and Gr1^+^ neutrophils significantly decreased in the DTR-M group compared with the WT, DTA, and DTR-S groups, while no significant difference was observed among the three groups (**Figures 1E–G**).

**Figure 2:**
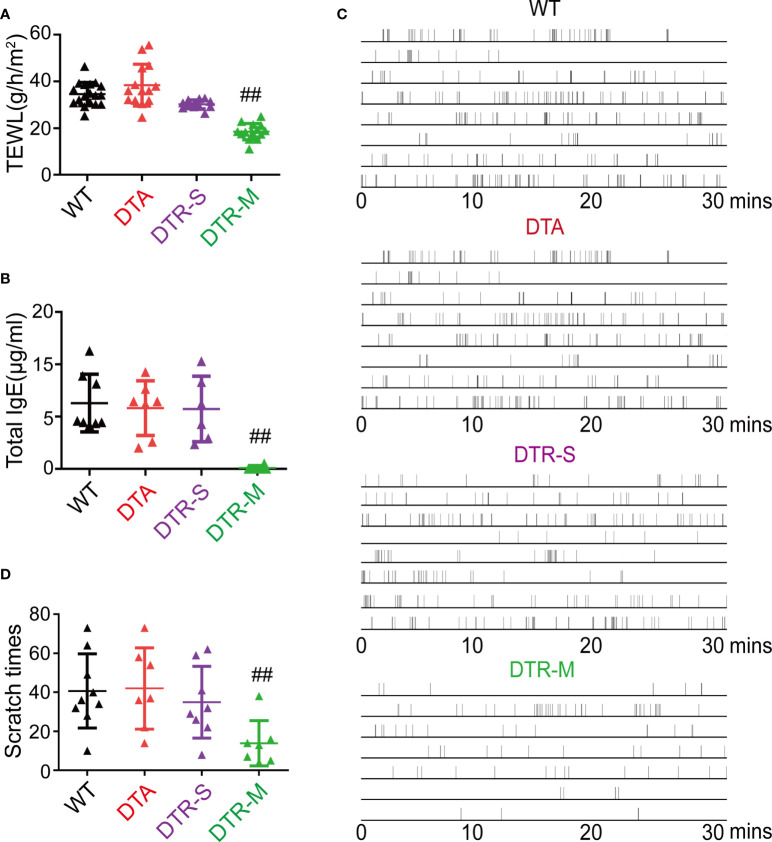
Depletion inflammatory Langerin^+^ dDCs significantly alleviate key features of MC903-induced AD-like dermatitis. **(A)** Trans-epidermal water loss (TEWL) of MC903-treated ears at day 14. **(B)** The total IgE level at day 14. **(C)** Time-table of scratching behavior (one horizontal line represents one mouse, each vertical line indicates one scratching) of mice. **(D)** Number of scratching behavior within 30 minutes in each group. One out of at least three representative experiments is depicted (mean ± SEM of n ≥ 3 mice per group). ^##^ *p* < 0.05 vs. WT, DTA and DTR-S groups. DTR-S, DTR mice with DT injection for one time; DTR-M, DTR mice with DT injection for multiple times.

Skin barrier impairment, elevated IgE, itching behavior, and percutaneous sensitization are the hallmarks of AD (15). The level of transepidermal water loss (TEWL), which is an indicator to reflect the skin barrier function, significantly decreased in the DTR-M group than in the other three groups (**Figure 2A**). The depletion of all skin Langerin^+^ cells in the DTR-M group resulted in nearly undetectable total serum IgE collected on day 15 (**Figure 2B**). Pruritus behavior for 30 min was recorded on day 15, and the total scratch times indicated that the DTR-M group displayed a less scratch behavior compared with the other three groups (**Figures 2C, D**). Interestingly, we found that continuous scratching, which meant bouts of scratching within 10 s, was common in the WT, DTA, and DTR-S groups (**Figure 2C**), which disappeared in the DTR-M group. Such continuous scratching mirrored the “itch-scratch cycle” in patients with AD. No significant differences in the levels of TEWL and total serum IgE and scratch behaviors were observed among the WT, DTA, and DTR-S groups irrespective of the existence of LCs/r-Langerin^+^ dDCs. Collectively, our results showed that only i-Langerin^+^ dDCs depletion can dramatically alleviate the inflammation, indicating that i-Langerin^+^ dDCs are critical for the MC903-induced AD-like dermatitis, while both LCs and r-Langerin^+^ dDCs are dispensable for this response.

### Depletion of i-Langerin^+^ dDCs prevent OVA-percutaneous sensitization

**Figure 3:**
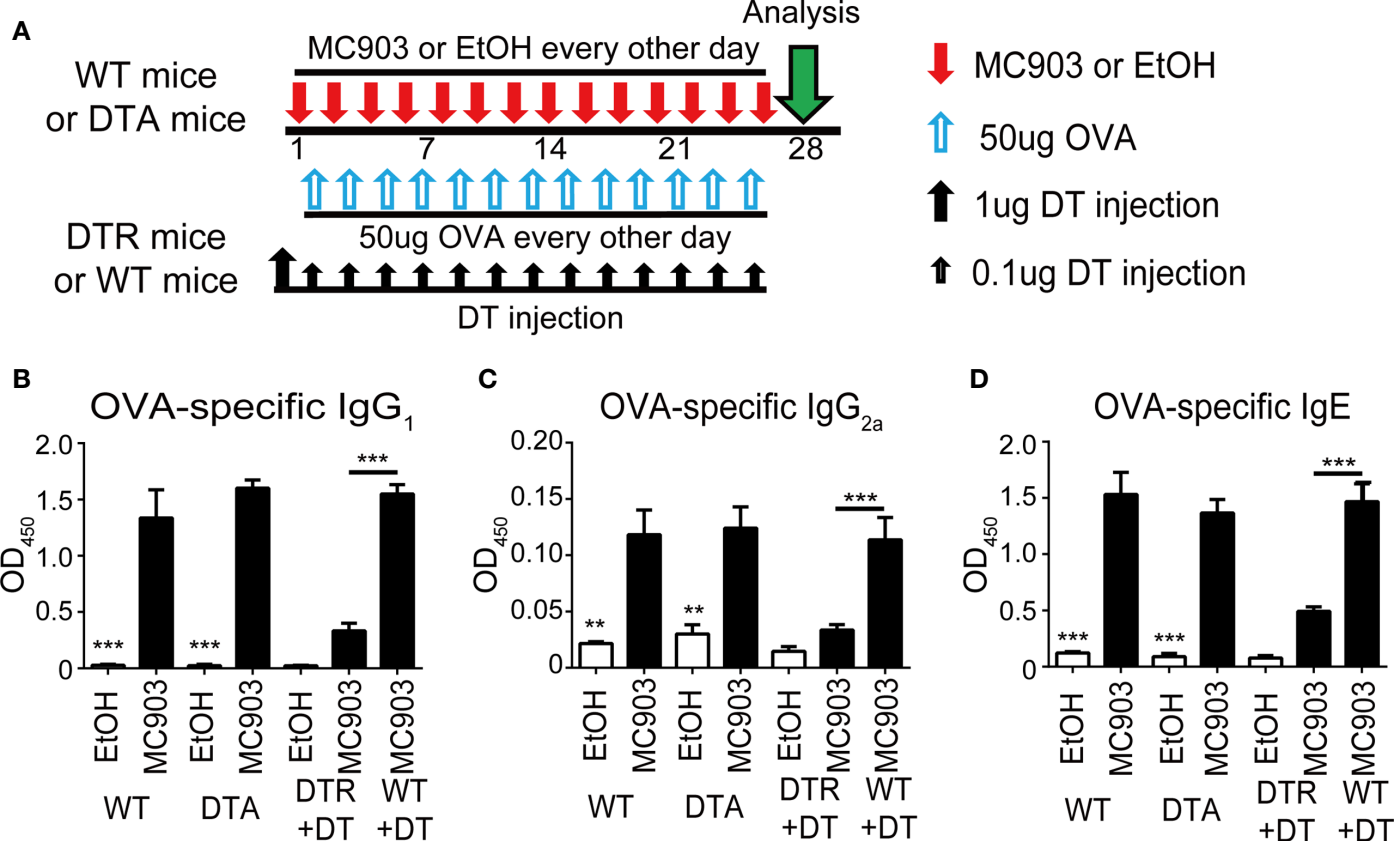
Inflammatory dermal Langerin^+^ DCs are necessary for OVA-percutaneous sensitization. **(A)** Strategy for percutaneous OVA sensitization. **(B–D)** Absorbance of OVA-specific immunoglobulins IgG_1_, IgG_2a_, and IgE after percutaneous sensitization in each group. One out of at least two representative experiments is depicted (mean ± SD of n ≥ 5 animals per group). EtOH, ethyl alcohol; OVA, ovalbumin. **p < 0.01; ***p < 0.001.

Percutaneous sensitization is believed to be a major route for allergen-specific IgE generation. MC903 and ovalbumin (OVA) were separately applied to the ears of mice to induce the production of OVA-specific antibodies to characterize the specific role of LCs and r-/i-Langerin^+^ dDCs in the production of allergen-specific antibodies (**Figure 3A**). The serum was harvested on day 28 for enzyme-linked immunosorbent assay (ELISA) of OVA-specific IgG1/IgE and the Th2 cell-associated isotype IgG2a. The results showed that only the mice in the DTR-M group lacking i-Langerin^+^ dDCs displayed markedly decreased OVA-specific IgG1/IgE and IgG2a levels compared with the other groups. DTA mice and DTR-S mice, lacking LCs and r-Langerin^+^ dDCs, showed no significant difference compared with each other and WT mice. The interference of DT with the results was excluded because no difference was noted between WT mice with and without DT injection (**Figures 3B–D**). Our results suggest that i-Langerin^+^ dDCs, instead of LCs or r-Langerin^+^ dDCs, are required to mediate OVA-specific percutaneous sensitization.

### i-Langerin^+^ dDCs facilitated TSLP production in the early stage of MC903-induced AD-like dermatitis

The initiation of the MC903-induced murine model of AD is dependent on abundant TSLP production and TSLP-responsive DCs. Previous studies showed that DCs not only responded to TSLP but also produced TSLP (16, 17). Therefore, we investigated whether the TSLP production was affected when LCs or/and r-/i-Langerin^+^ dDCs were absent in the initial stage of this AD model.

**Figure 4:**
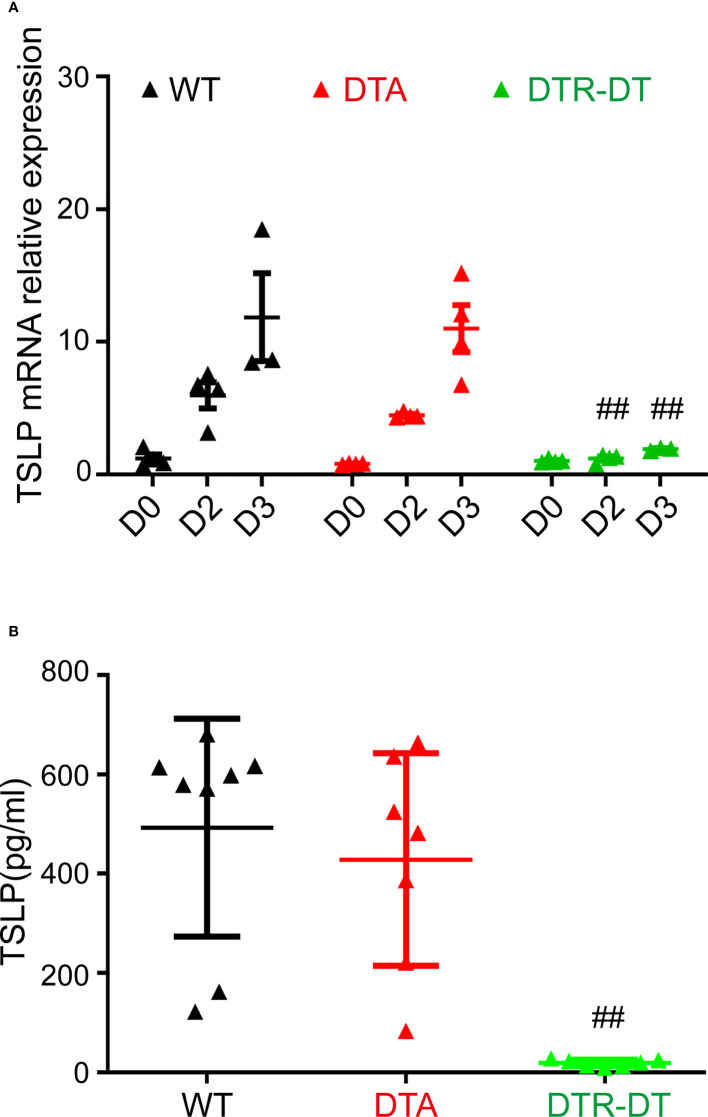
Inflammatory dermal Langerin^+^ DCs facilitate TSLP production in the initiation stage of MC903-induced atopic AD-like dermatitis. **(A, B)** DTA mice, DTR mice after DT injection (DTR-DT) and wild type (WT) mice were topically applied daily with 2 nmol MC903. Ear tissues were harvested at Day 0 (D0, untreated ear), D2 and D3, serum were collected at day 3. **(A)** Relative TSLP mRNA expression of ears at indicated time points, mean expression was shown above each column. **(B)** The serum TSLP level was measured at day 3. One out of at least three representative experiments is depicted (mean ± SEM of n ≥ 3 mice per group). p< 0.05 vs. WT, DTA and DTR-S groups. ^##^p < 0.01.

MC903 was applied to the ears of WT mice, DTA mice (only lacking LCs), and DTR mice with DT treatment (lacking both LCs and r-/i-Langerin^+^ dDCs within3 days) to induce AD. The ear tissues were harvested on day 0 (before treatment), day 2, and day 3 for qRT-PCR analysis. The serum of peripheral blood was collected on day 3 for ELISA. The qRT-PCR results showed that the mice exhibited a nearly undetectable mRNA level of TSLP before MC903 treatment. Shortly after MC903 treatment, the levels of TSLP mRNA in WT and DTA mice rapidly increased on days 2 and 3 in a time-dependent manner (**Figure 4A**). Surprisingly, TSLP mRNA expression significantly reduced in DTR mice with the deficiency of both LCs and dermal Langerin^+^ DCs after DT injection (**Figure 4A**). The ELISA results further confirmed that the levels of serum TSLP were almost undetectable in DTR mice, but equally high in WT and DTA mice on day 3 (**Figure 4B**). Consistence with previous findings, the levels of several other AD-associated chemokines and cytokines were almost unchanged on day 3, including CXCL2, IFN-γ, IL-10, IL-13, IL-1β, IL-4, IL-6, and TNF-α (**Supplemental Figure S1A**).

DCs can also produce TSLP in response to allergens (18). Therefore, the reduction of TSLP production in DT-injected DTR mice may attribute to the absence of TSLP production by LCs and dermal Langerin^+^ DCs themselves. Thus, all Langerin^+^ cells in the atopic ear skin of Langerin-EGFP mice were sorted to determine the mRNA expression of TSLP. The results showed that TSLP expression increased rapidly in both Langerin^+^ cells (including LCs and dermal Langerin^+^ DCs) and Langerin^-^ cells (mainly keratinocyte cells) after twice MC903 treatment (**Supplemental Figure S2A**). Interestingly, the magnitude of TSLP expression in Langerin^+^ cells was comparable to that in Langerin^-^ cells (mainly keratinocytes), indicating that Langerin^+^ DC cells and keratinocyte cells were important contributors for TSLP production. Combined with the null role of r-Langerin^+^ dDCs in the inflammation, these results indicated that i-Langerin^+^ dDCs might facilitate the TSLP production in the early stage of MC903-induced AD-like dermatitis.

### TSLP facilitates the generation of BM-derived Langerin^+^ DCs by upregulating Langerin expression *in vitro*

**Figure 5:**
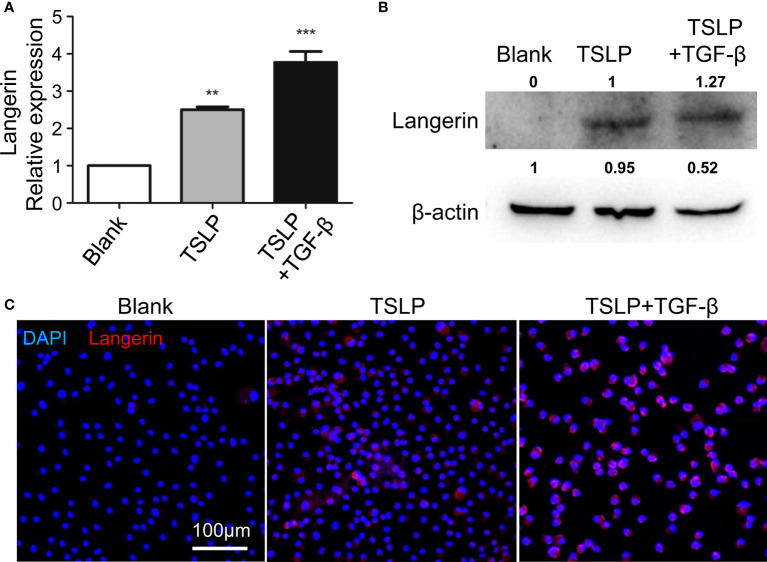
TSLP promotes generation of BM-derived Langerin^+^ DCs by upregulating Langerin expression *in vitro*. BM cells were prepared from WT mice, and were incubated with GM-CSF, IL-4, TSLP, and with or without TGF-β to produce BMDCs. At day 9, BMDCs were harvested and the expression of Langerin were analyzed by qRT-PCR **(A)**, Western Blot **(B)** and Immunofluorescence **(C)**. scale bars represent 200 μm. n = 5, data shown are mean ± SEM, and representative of at least three independent experiments. **p < 0.01; *** p< 0.001.

Upon inflammation, the BM cells can transform into tissue-specific DCs and exert important roles. In this study, the discrepancy between DTR-M and DTR-S groups was the existence of newly recovered i-Langerin^+^ dDCs, which gave rise to two kinds of completely contradictory results of TSLP production. Langerin upregulation was important for the transformation of Langerin^+^ DCs. Previous studies demonstrated that TSLP cooperated with TGF-β to promote the expression of Langerin in human blood DCs. We examined whether TSLP promoted the formation of Langerin^+^ DCs by increasing Langerin expression on DCs in mice. BM-derived DCs **(**BMDCs) were produced from WT mice by stimulating the BM cells with IL-4, GM-CSF, TGF-β, and/or TSLP. Then, BMDCs were harvested to detect the expression of Langerin by qRT-PCR, Western Blot, and immunofluorescence analysis. The results showed that, compared with the controls, the expression level of Langerin on BMDCs significantly increased upon TSLP stimulation and further increased upon the combined stimulation of TSLP and TGF-β (**Figures 5A–C**). These data indicated that TSLP could promote the generation of BM-derived Langerin^+^ DCs *in vitro*. Therefore, it was likely that high levels of TSLP in the microenvironment of AD transformed the newly recruited DCs into Langerin^+^ DCs, and this population of DCs in turn promotes the inflammation. The formed loop assists and maintains the development of AD.

### BM-derived i-Langerin^+^ dDCs are the principal contributors to the MC903-induced inflammation

**Figure 6:**
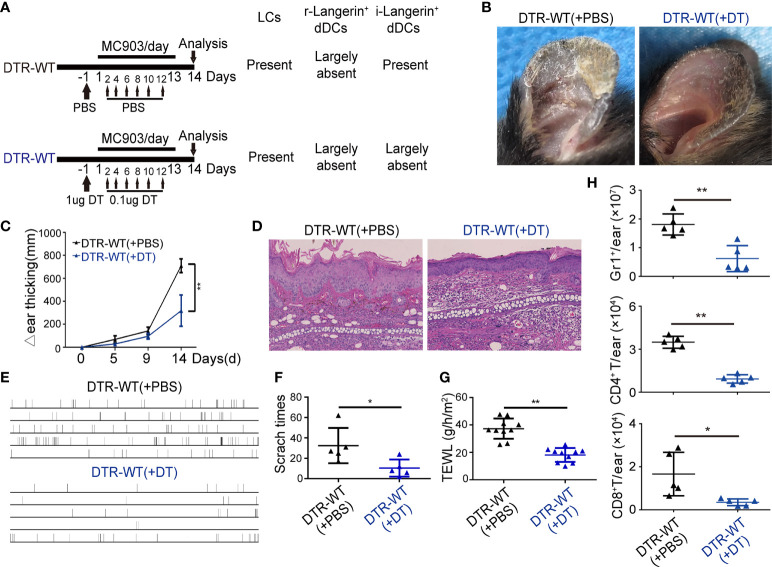
BM derived inflammatory dermal Langerin^+^ DCs alleviated the MC903-induced inflammation. BM cells of DTR mice were transplanted to γ-irradiated WT mice. After six weeks, the chimeric mice (DTR-WT) treated with DT or PBS injection were painted with MC903 on the ears for 13 days. **(A)** Strategy for treatment. **(B)** Gross appearance of MC903-treated ear at day 14. **(C)** Dynamic change of ear thickness. **(D)** Representative H&E-stained ear skin sections. Scale bar = 50 μm. **(E)** Time-table of scratching behavior of each chimeric mice. **(F)** Number of scratching behavior within 30 minutes in each group. **(G)** Trans-epidermal water loss (TEWL) of MC903-treated ears at day 14. **(H)** The absolute numbers of Gr1^+^ neutrophils, CD4^+^ T or CD8^+^ T cells per ear. One out of at least three representative experiments is depicted (mean ± SEM of n ≥ 3 mice per group). ^*^ *p* < 0.05. **p < 0.01.

Using different DT injection strategies in DTR mice, our data demonstrated that only depletion of i-Langerin^+^ dDCs could result in significantly alleviated inflammation. The chimeric mice (DTR-WT) were constructed by transplanting DTR BM cells into lethally irradiated congenic WT recipients to directly address the role of i-Langerin^+^ dDCs in AD. Because of the irradiation-resistant characteristics of LCs, r-Langerin^+^ dDCs were ablated and other DC subsets, including i-Langerin^+^ dDCs, in the DTR-WT chimeric mice were reconstituted from the transplanted BM, which could be depleted after DT treatment (**Figure 6A**). MC903-induced AD-like dermatitis was produced after 6 weeks of transplantation. No significant inflammation was observed in WT mice with the existence of Langerin^+^ DCs and PBS-treated DTR-WT chimeric mice only lacking r-Langerin^+^ dDCs. However, DTR-WT mice with DT injection, lacking r-Langerin^+^ dDCs and BM-derived i-Langerin^+^ dDCs, demonstrated significantly alleviated skin inflammation, as evidenced by an obvious improvement in gross appearance (**Figure 6B**), decreased ear thickness (**Figures 6C, D**), decreased level of TEWL, less scratch behavior (**Figures 6E–G**) and decreased number of infiltrating Gr1^+^, CD4^+^ and CD8^+^ cells (**Figure 6H**). Hence, these results demonstrated that BM-derived i-Langerin^+^ dDCs promoted the development of MC903-induced AD-like dermatitis.

## Discussion

The contributions of distinct DC subsets in AD are poorly understood. In the present study, we used DTA and DTR mice as well as BM transplantation, and showed that the absence of BM-derived i-Langerin^+^ dDCs could attenuate the MC903-induced AD-like dermatitis, which was indicated by alleviated symptoms, less scratch, low level of serum IgE, and nearly undetectable TSLP expression. The influx of i-Langerin^+^ dDCs into the lesions augmented local TSLP production either by keratinocytes or by themselves. The *in vitro* experiments revealed that TSLP upregulated the expression of Langerin on BMDCs, suggesting that a high level of TSLP in local atopic lesions could further facilitate the formation of BM-derived i-Langerin^+^ dDCs.

DCs are a heterogeneous population of cells in the immune system, and LCs are the DC subset residing in the suprabasal layers of the epidermis. In 2007, r-Langerin^+^ dDCs were discovered and termed a new DC subset distinguished from LCs (14, 19, 20). Subsequent studies uncovered the different functions of LCs and r-Langerin^+^ dDCs from several aspects of skin homeostasis (21, 22). Previous conclusions on the role of LCs in the pathogenesis of AD are controversial. By using DTA mice and DTR mice, we found that LCs were dispensable for MC903 AD mouse model. This is in agreement with the previous findings conducted by Lee et al. (23). They demonstrated that dermal DCs, but not LCs, are critical in single epicutaneous sensitization in both C57BL/6 mice and BALB/c mice (23). The discrepancy between us and this report is that we combine DT injection strategy and determine i-Langerin^+^ dDCs, but not r-Langerin^+^ dDCs, are essential for the pathogenesis of AD. In contrast, another study revealed a dual function of LCs in TSLP-promoted Tfh/Th2 differentiation that promoted Tfh differentiation in MC903-AD mouse model, and inhibited Tfh/GC response as well as suppressed Th2 skin inflammation and the atopic march in OVA-AD mouse model (24). Saeko et al. applied DTA mice to an OVA-induced epicutaneous sensitization model and reported that LCs initiate epicutaneous sensitization with protein antigens and induce Th2-type immune responses *via* TSLP signaling (25). The discrepancy among the research groups could explain by some factors, such as the allergen application method: topical OVA versus long exposure of OVA; the concentration of MC903, the difference of mouse background: BALB/c versus C57BL/6; or the site of allergen application: ear versus back, or maybe some factors yet to be determined. In the present study, strict designs were performed to distinguish the roles of LCs, r-Langerin^+^ dDCs, and i-Langerin^+^ dDCs using DTA mice, DTR mice with single/multiple DT injection, and BM transplantation. The comprehensive results showed that i-Langerin^+^ dDCs, but not LCs or r-Langerin^+^ dDCs, were essential for the pathogenesis of AD. Both r-Langerin^+^ DCs and i-Langerin^+^ DCs are originated from BM and radiation sensitive, but there remain some differences. The r-Langerin^+^ DC subset are formed during embryo development and exist in the steady state. And the r-Langerin^+^ DC subset derived from BM upon inflammatory stimulation. Previous study showed that the phenotype of r-Langerin^+^ DCs is Langerin^+^MHC class II^+^CD11c^+^CD11b^low^EpCAM^–^CD103^+^ (26). Based on our observations and their function, we speculate that the different phenotype of i-Langerin^+^ DCs is highly expressed CD11b and F4/80, which needs to be determined by further studies.

TSLP is highly expressed by keratinocyte cells in patients with AD and plays an important role in this disease. TSLP can induce an innate phase of Th2 response by indirectly activating DCs, mast cells, and innate lymphoid cells to produce Th2 cytokines and chemokines, or directly by reprogramming transcriptionally in mouse CD4^+^ T cells to increase the number of IL-4-, IL-5-, and IL-13-producing cells. DCs and TSLP-responsive DCs were found to be important in the induction of Th2 immune response. Previous findings showed that DCs not only responded to TSLP but also produced TSLP (5, 16, 18). We also found that CD11c^+^ Langerin^+^ DCs could produce TSLP during the initiation of AD. However, DCs could not be a major source of TSLP due to their small proportion. DCs have been known as antigen-presenting cells. A more recent study by Florent Ginhoux showed that skin cDC1s were essential regulators of the innate response in cutaneous immunity and had roles beyond classical antigen presentation (27). Our findings also suggested that i-Langerin^+^ DCs could act as a regulator to promote the production of abundant TSLP by keratinocytes; however, the mechanisms need further exploration. The early production of TSLP preceded the infiltration and recruitment of DCs expressing its receptor TSLPR and then activated DCs to provide a Th2-polarizing microenvironment by upregulating OX40L (28). TSLP-mediated activation of DCs triggered CCR7-dependent migration to the draining lymph nodes and enhanced their capacity to initiate Th2 responses. However, Th2 cytokines abrogated the induction of CCR7 mediated by TSLP, implying that TLSP-activated CD1c^+^ DCs were retained in the inflamed tissue during a Th2-mediated inflammatory reaction. The mouse model of AD used in this study was dependent on abundant TSLP and its responsive DCs. Thus, it was possible that the local high level of TSLP stimulated the infiltrated DCs retained by Th2 cytokines and promoted the transformation of some DCs with upregulated Langerin expression into another subset of Langerin^+^ DC, as evidenced by our findings that TSLP could facilitate Langerin expression on BMDCs. These new i- Langerin^+^ DCs in turn promoted TSLP production and inflammation. BDCA-1^+^ myeloid DCs were the principal DCs in atopic lesions in patients with AD. A previous study confirmed that human blood BDCA-1^+^ DCs differentiated into LCs-like cells by promoting Langerin expression with TSLP and TGF-β. This population had a mature phenotype and the potential to polarize CD4^+^ T cells into Th2 cells, which was concordant with our observation in BM-derived DCs of mice (29). We found that TSLP could promote the expression of Langerin on BMDCs and further increased the level in combination with TGF-β. At present, the difficulty in performing research on Langerin^+^ DCs research is the small number of cells and the lack of cell lines. This study might provide a new way to induce mouse Langerin^+^ DCs, including LCs, by stimulating BMDCs with TSLP and TGF-β. More characteristics such as Birbeck granules are needed to identify the mouse Langerin^+^ DCs induced in this way, which can be ascertained to be LCs.

This study had several limitations. First, the phenotype of i -Langerin^+^ dDCs could not be identified. Second, the conclusion that TSLP could promote Langerin expression on BMDCs and favor the formation of newly infiltrating Langerin^+^ DCs was not confirmed *in vivo*. Third, the MC903-induced AD mouse model could not recapitulate but only reflected limited aspects of human AD. AD has a complex etiology that involves abnormal immunological and inflammatory pathways, including defective skin barriers, exposure to environmental agents, as well as neuropsychological factors. Although the topical application of MC903 to mouse skin recapitulates the features of AD, such as inflammation, itching, barrier dysfunction, and elevated serum IgE, the generation of skin inflammation does not require the presence of mature B and T lymphocytes, which are the effector cells driving AD progression (30). MC903 can upregulate epidermal TSLP expression, which may result in immune modification. Besides, other factors, such as dosage and duration, have an important influence on stimulating ability.

In summary, this study provided evidence that the new infiltrated inflammatory Langerin^+^ DCs promoted the development of AD-like dermatitis by mediating the production of TSLP. Also, TSLP promoted Langerin expression on BMDCs and facilitated the formation of new infiltrated Langerin^+^ DCs. Our data highlighted the specific role of DC subsets in AD and further underlined the importance of TSLP in acting with DCs to promote Th2 immune response and Th2-mediated inflammatory diseases.

## Data availability statement

The original contributions presented in the study are included in the article/**Supplementary Material**. Further inquiries can be directed to the corresponding author.

## Ethics statement

The animal study was reviewed and approved by the guidelines of the Fourth Military Medical University.

## Author contributions

MF, CX, and ZZ designed the study, analyzed the data and wrote the paper; CX, ZZ, CZ, JG, YL, HF, HQ, and WL performed the experiments and analyzed the data; GW coordinated the experiment and reviewed the manuscript. All authors read the manuscript and contributed to the discussions and revision. All authors contributed to the article and approved the submitted version.

## Funding

This work was supported by grants from the National Natural Science Foundation of China (nos. 82173409 and 82003341) and Shaanxi scientific research grant (nos. 2022ZDLSF03-14).

## Acknowledgments

We thank our colleagues and collaborator for their support.

## Conflict of interest

The authors declare that the research was conducted in the absence of any commercial or financial relationships that could be construed as a potential conflict of interest.

## Publisher’s note

All claims expressed in this article are solely those of the authors and do not necessarily represent those of their affiliated organizations, or those of the publisher, the editors and the reviewers. Any product that may be evaluated in this article, or claim that may be made by its manufacturer, is not guaranteed or endorsed by the publisher.
