# Supplementary figures and images for: A population of dermal Langerin+ dendritic cells promote the inflammation in mouse model of atopic dermatitis
Source: Front Immunol. 2022 Oct 3;13:981819. doi: 10.3389/fimmu.2022.981819 (PMC9592551; doi:10.3389/fimmu.2022.981819)

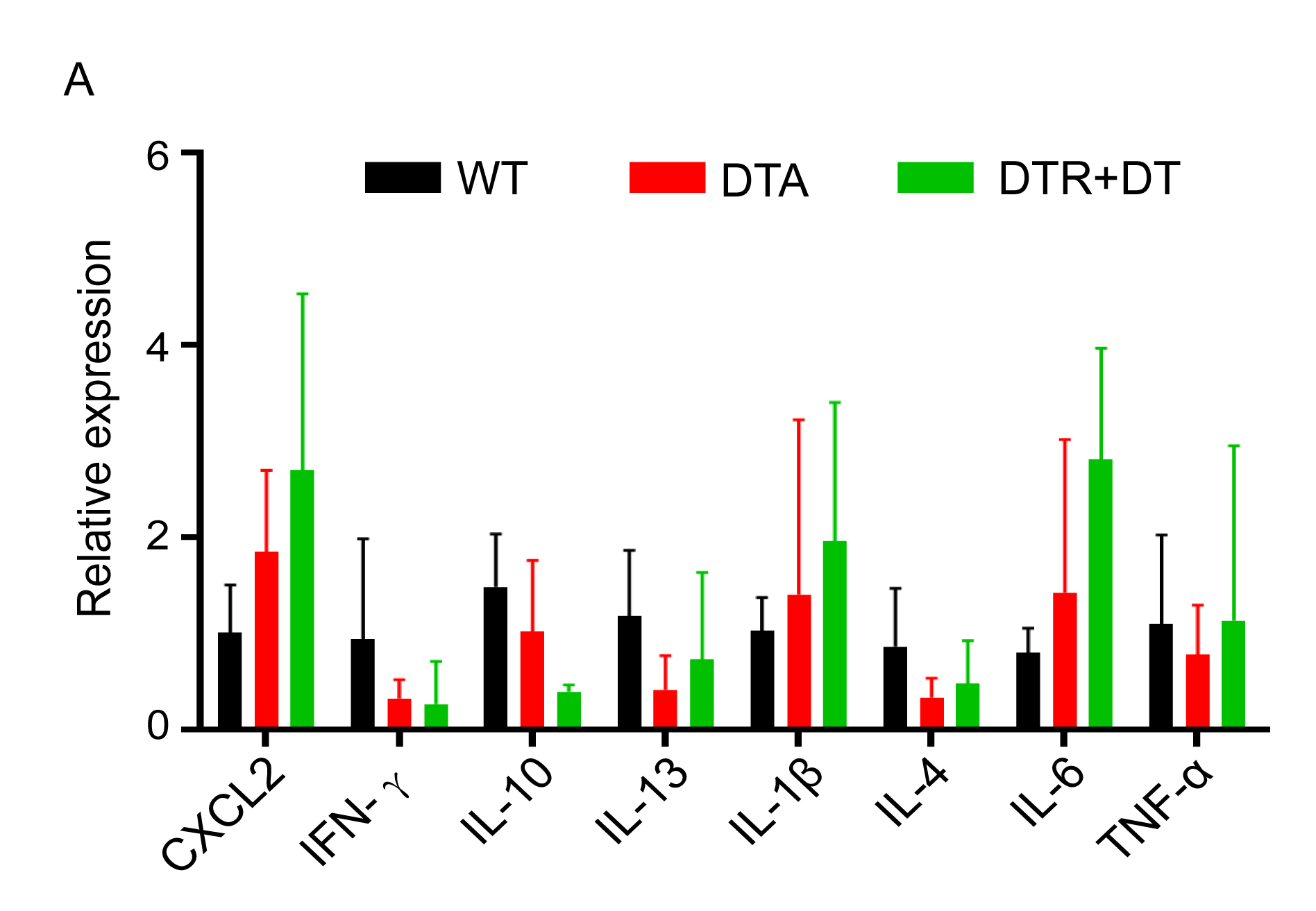

Supplement: Supplementary file 1 [file Image_1.tif]

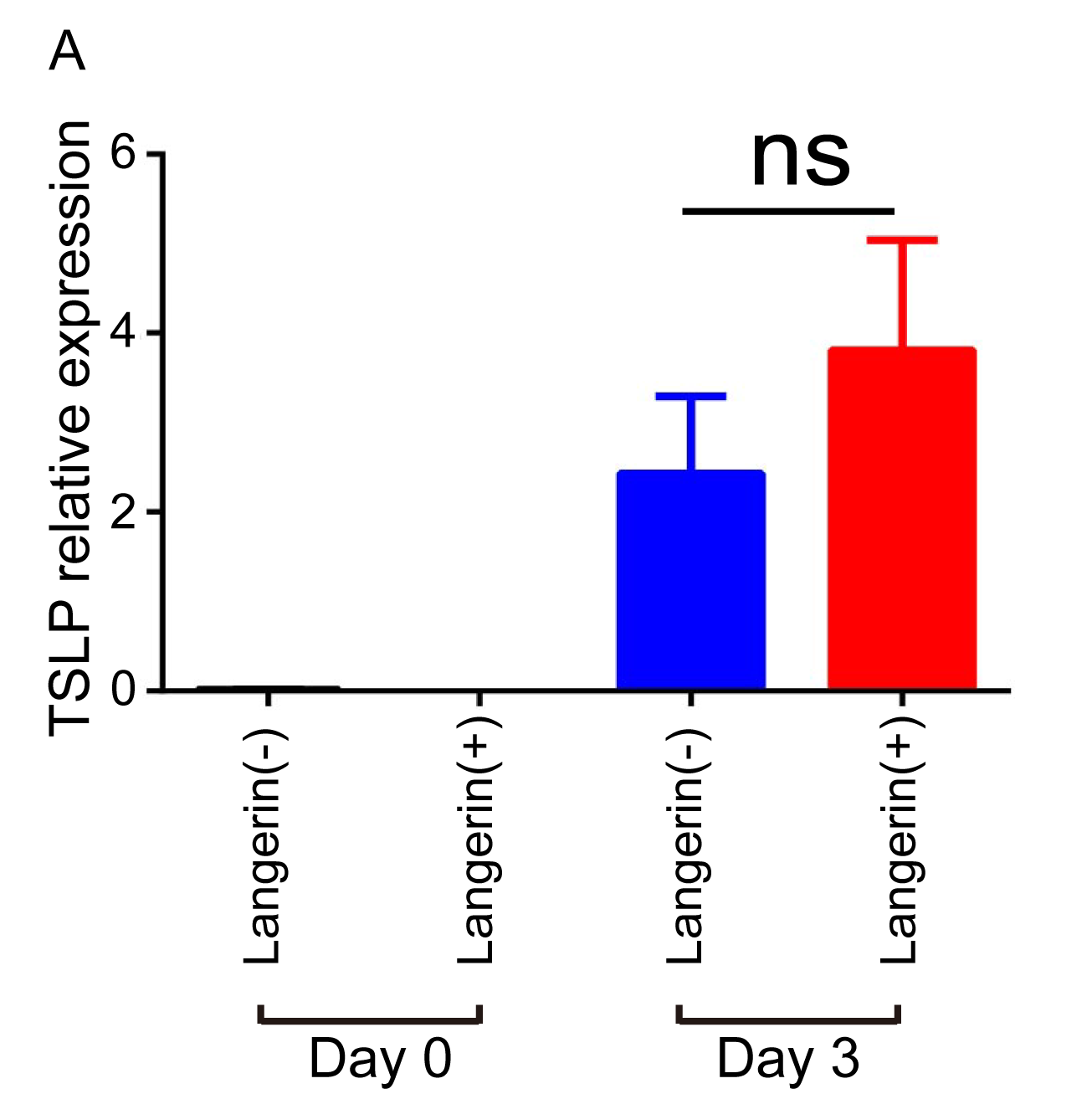

Supplement: Supplementary file 2 [file Image_2.tif]
